# Association between Actual and Perceived Motor Competence in School Children

**DOI:** 10.3390/ijerph17103408

**Published:** 2020-05-14

**Authors:** Jaime Carcamo-Oyarzun, Isaac Estevan, Christian Herrmann

**Affiliations:** 1 Department of Physical Education, Faculty of Education, Social Science & Humanities, Universidad de La Frontera, 4811230 Temuco, Chile; 2 Activitat Física i Promoció de la Salut (AFIPS) Research Group. Department of Teaching of Music, Visual and Corporal Expression, University of Valencia, 46022 Valencia, Spain; isaac.estevan@uv.es; 3 Didactics Exercise and Sport, Zurich University of Teacher Education, CH-8032 Zurich, Switzerland; christian.herrmann@phzh.ch

**Keywords:** motor development, fundamental movement skills, self-perception, gender

## Abstract

*(1) Background:* The association between actual and perceived motor competence (MC) is one of the underlying mechanisms that influence the practice of physical activity. This study mainly aimed to analyze the structure and correlations between actual and perceived MC in schoolchildren and to compare actual and perceived MC between girls and boys. *(2) Methods:* A total of 467 fifth and sixth graders (43.9% girls, *M* = 11.26, *SD* = 0.70) participated. Actual and perceived MC were assessed. To examine the proposed four factor models, structural equation models (factor analyses, latent correlations, invariance testing for gender) were conducted. Student t-test for independent samples was used to compare boys and girls. *(3) Results:* Proposed models achieved acceptable fit values with moderate correlation between the factors according to the type of MC in actual and perceived MC. Invariant factor structure in boys and girls was revealed. Boys performed and perceived themselves higher in object control than girls; whereas girls showed higher actual and perceived self-movement than boys. *(4) Conclusions:* The association between actual and perceived MC exists both globally and separately by gender, despite the differences between boys and girls. It is important to consider the role of gender and type of MC in the development of motor competencies, as well as in the strengthening of the children’s sense of competence.

## 1. Introduction

The development of appropriate levels of motor competence (MC) during childhood is considered as an essential factor for the adherence to the practice of physical activity (PA) [1,2] as well as for its promotion throughout life span [3]. This is why it is recognized as one of the preponderant factors for the consolidation of physical literacy [4]. MC is a functional performance disposition that permits the children to meet concrete situation-specific demands in the culture of sports and exercise [5]. The realization of these motor demands is reflected through the performance of certain fundamental motor skills (FMS), which are the basis for an individual’s future motor performance [6]. From a pedagogical perspective (school-based PE setting), these FMS are divided into two categories (or what can be considered as well as motor competencies): (a) self-movement, which is the movement and control of the body in an open space (running, galloping, jumping, etc.); (b) object control, which implies the use of hands and feet to handle objects (throwing, catching, bouncing, dribbling) [7]. If these FMS are not developed properly during childhood, it will be more difficult to learn more specific skills that allow more complex movements, which translates into a certain limitation for the children regarding their opportunities to participate in regular physical activities [1,2,3].

The importance of MC during the initiation, continuation or abandonment of regular PA is an emerging issue that has generated great scientific interest during the last decade, confirming the positive relation between MC and PA [8,9,10]. Thus, it has been conceptualized that the reciprocal and dynamic relation of development between MC and PA is measured by factors such as health-related physical fitness and perceived MC during childhood [1,2]. The consideration of perceived MC as an intervening and mediating factor between the actual MC and PA of children has led to the need to focus on the study of the relationship between actual and perceived MC during childhood. The emerging evidence shows that the actual MC interacts with the perceived MC, generating one of the most powerful underlying mechanisms that affect the commitment and persistence regarding the practice of PA [11,12,13].

Perceived MC refers to an individual’s perception of his or her own ability of performing certain motor skills [2] or the self-perception with respect to the actual MC [14]. The children with higher levels of perceived MC would show a higher willingness to participate in physical activities and persist in tasks that may be perceived as challenging [2,15,16]. Therefore, the perceived MC might be an important predictor regarding the levels of PA [11], because when children do not believe that they have the skills to perform a task, they are more likely to avoid participating [2].

Different degrees of association between actual and perceived MC have been evidenced in several studies [17,18,19,20,21,22,23], considering body mass index (BMI) and gender as some of the determining factors. The role of the BMI is clearer than the role of gender. There is evidence that suggests an inverse relationship between MC and BMI, which is incipient in preschoolers [24,25,26] and can be confirmed in primary school [27,28]. Children with overweight or obesity have more problems with physical activities that involve controlling the body mass compared to children of normal weight [29,30]. An increased mass makes it more difficult to stabilize and/or propel the body, resulting in a lower actual and perceived MC, which decreases the likelihood that people who are overweight or obese will engage in PA [13,31]. Children who are overweight also have significantly lower levels of perceived MC [13,32,33,34]. This low perception is related to a poor body image, because overweight children are perceived as not exercising, lazy, less athletic and fit, and had overall a lower self-image than children with normal weight [35,36,37]. On the other hand, the role that gender plays as a correlate of MC is not entirely clear, mainly because the results are different regarding the type of FMS [38,39]. In the case of actual MC, most studies indicate that boys show significantly better levels of competence regarding object control [38,40]; however, in actual self-movement, there is no clear consensus [38], since some studies indicate that girls perform better than boys [21,41], while others could find no differences between genders [42,43,44]. In terms of perceived MC, the differences between genders seem to increase during childhood development, with no differences found between preschool boys and girls [45,46], while boys in primary school indicate a higher perceived MC regarding object control than girls [43,47]. These differences between genders cannot be found regarding self-movement [43]. Regarding the relationship between actual and perceived MC, there is evidence that girls underestimate their actual object control skills, while boys overestimate themselves [22]. Longitudinal studies also present differences between gender, confirming that the type of FMS would influence the association between the actual MC and perceived MC [48,49]. The development of object control skills significantly increases from preschool to primary school, being more marked in boys, while the self-movement skills do not present any significant increase, although the levels of girls are higher in preschool and primary school [48]. 

Although the existing evidence confirms the reciprocal relationship between actual and perceived MC, the results according to gender are very heterogeneous, possibly because most studies conduct measurements with instruments that limit the alignment between actual and perceived MC and that possibly measure different MC domains [14]. Therefore, it is pertinent to study the relationship between actual and perceived MC using measuring methods focused on the same specific domain, by elaborating a structural model that presents these relationships in a comprehensive way, but also by analyzing if the relationships of this model are present in girls and boys. Within this framework, the main objective of the study is to analyze the structure and relationship between actual and perceived MC in school-age children according to gender and to compare the actual and perceived MC between girls and boys. A second purpose is to analyze the association of actual and perceived MC with BMI according to the type of MC. Based on the theoretical framework presented in this introduction, it has been hypothesized that for both actual and perceived MC, according to the type of FMS, two factors corresponding to object control and self-movement would be found, which are significantly related to each other independent of gender. Moreover, using BMI would be associated with actual and perceived MC regardless of the type of MC.

## 2. Materials and Methods 

### 2.1. Sample

A convenience sample of 467 schoolchildren (43.9% girls) aged between 10 and 13 years (girls M = 11.16 SD = 0.69, boys M = 11.34 SD = 0.70, total M = 11.26 SD = 0.70) of fifth and sixth grade of primary school, from seven schools in the La Araucanía Region in Chile participated in this study. Although it is a non-probability sample, a method to represent the different types of school in Chile was used. That is, the sample was determined through the draw of schools, considering the proportions of each type of school: public (2 schools), subsidized (4 schools) and private (1 school). 

### 2.2. Instruments

#### 2.2.1. Actual MC

The MOBAK 5-6 test instrument (MOBAK = acronym of MOtoriche BAsisKompetenzen in German) developed by Herrmann and Seelig [5] and validated in Spanish by Carcamo-Oyarzun and Herrmann [50], was used to assess the actual MC. This instrument includes a total of eight test items (Table 1), four for the object control competence (throwing, catching, bouncing and dribbling) and four for the self-movement competence (balancing, rolling, jumping, and running). This two-factor structures (object control and self-movement) previously established in the original version [5] and in the Spanish version [50] of the MOBAK 5-6 test were considered as two latent factors.

Each of the items of the tests are marked on a dichotomous scale (0 = failed, 1 = successful), recording the number of successful attempts (no successful attempts = 0 points, one successful attempt = 1 point, two successful attempts = 2 points). The children had two attempts at each item, with exception of the test items throwing and catching. For these two items, the children had six attempts each, 0–2 successful attempts were scored as 0 points, 3–4 successful attempts as 1 point, and 5–6 successful attempts as 2 points. The procedures for performing and marking the tests are described in the MOBAK manuals (see details in Herrmann and Seelig [51,52]). 

#### 2.2.2. Perceived MC 

The SEMOK (an acronym of SElbstwahrnehmung MOtorischer Kompetenzen in German) questionnaire of Herrmann and Seelig [19] was used. It was translated into Spanish by the reverse translation method, without any change in the content of the questionnaire. This questionnaire is designed for the assessment of perceived MC in children in grades 5 and 6 of primary school and refers directly to the MOBAK 5-6 test instrument. This means that the SEMOK questionnaire also has 8 items, divided into two-factor structures (object control and self-movement). Like the original version of the SEMOK questionnaire [19], these two-factors were considered as two latent factors.

The eight SEMOK items refer to the children’s assessment of whether they are capable of meeting the basic motor demands (e.g., throwing, catching, balancing, rolling). The schoolchildren were asked to indicate to what extent they consider themselves capable of performing these motor tasks (Table 2), by answering the question, “Do you think you can do the following activities?” For example, in relation to the task of jumping in the MOBAK 5-6 test, in which the child jumps the rope for 20 s, changing the rhythm after 10 s [5], the item in the SEMOK questionnaire is “I can jump the rope and change the rhythm”. In addition to the verbal description, all items are accompanied by a pictographic description of the motor task. The response format consists of a Likert scale from 1 to 5, on which the schoolchildren express their degree of agreement with the statement made in each item (1 = totally disagree, 5 = totally agree).

#### 2.2.3. Body Mass Index (BMI)

Height and weight were measured to determine the BMI (kg/m^2^) as a covariate of the motor competencies. Height was measured using a SECA 213 stadiometer and weight was measured using an electronic calibrated scale TANITA UM2204. The mean BMI of the children was 21.92 kg/m^2^ (SD = 4.11). Due to the low correlations of the BMI with age (*r* = 0.10, *p* = 0.05) and gender (*r* = −0.028, *p* = 0.58), we used the BMI-raw values for the analyses.

### 2.3. Procedures

The research protocol was evaluated and approved by the Scientific Ethical Committee of the Universidad de La Frontera according to Approval Act No.122_17. The parents or guardians of the participants signed an informed consent form, authorizing the participation of the child. At the same time, the schoolchildren signed an informed consent form to confirm their willingness to participate in the study. The data were collected during physical education class. Before the actual MC measurements were taken, a tester met with each group in a traditional classroom to present the objectives of the study, to explain what the SEMOK questionnaire consists of and how it has to be answered. The estimated time for answering the questionnaire was about 15 min for an entire group. Afterwards, the schoolchildren went to the gym, where eight testers, who are trained in the application of the MOBAK tests, carried out the measurements. Each of the testers was responsible for a group of three to five children. The tester went with his group from one test to the next, until each child of the group completed all tasks. At each station the tester explained how the motor task is performed and then demonstrated it. In accordance with the description of the instrument, each child performed two attempts (except for the throwing and catching tasks, where they had six attempts), without previous trial attempts. The approximate duration of the application of all MOBAK items was 45 min.

### 2.4. Data Analyses

The data processing, descriptive and difference analyses were calculated with SPSS v. 25 (IBM Corp, Armonk, NY, USA), while the multivariate analysis was performed with Mplus v.8.1 (Muthén & Muthén). 

To examine the latent correlations between the two latent factors of the MOBAK 5-6 test and the two latent factors of the SEMOK questionnaire, a confirmatory factor analysis with four latent factors (Model 1) was performed using the mean- and variance-adjusted weighted least squares (WLSMV) estimator [53]. Factor loadings and residual variance were estimated freely for each test item. By using the results of this confirmatory factor analysis, the factor reliability (FR) was calculated. FR is a measure of the reliability over the total sum of all test items that form a construct. It should take values preferably greater than 0.6 [54].

Furthermore, we realized a sequential procedure with two models in order to examine the measurement invariance of the model concerning gender. Missing measurement invariances would restrict the validity of the results on gender differences and hint at a presence of a differential item functioning (DIF). This involved taking two steps with increasingly stringent nested models [55,56,57]: configural invariance (Model 2) and factorial invariance (Model 3).

We examined the configural invariance in the multiple group model 2, which allowed a model test for girls and boys simultaneously. All parameters were estimated freely. Only the factor structure (amount and type of latent factors and loadings) was equated between boys and girls in order to make sure that the factor structure was the same for boys and girls.

Model 3 measured the factorial invariance, which restricted the relation between the items and the latent factors. We equated additional factor loadings concerning the boys and girls by constraining the non-standardized factor loading above boys and girls invariantly. The tests of gender-specific differences between the six inter-correlation of the four latent factors follow the Wald Test of Parameter Constraints in Mplus.

As model 2 and model 3 were nested, the models could be compared using a difference test. We performed this with the Mplus module chi-square difference testing for WLSMV-estimations [58]. 

Finally, in model 4, we conducted a confirmatory factor analysis with the covariate gender and BMI (MIMIC) based on model 1 in order to be able to estimate the differences in the latent factors between boys and girls and the association with BMI.

On account of the multilevel structure (children from different grades), we considered the dependencies within the multilevel structure in the models by correcting the standard error with the type = complex function for nested datasets implemented in Mplus. The RMSEA (Root Mean Square Error of Approximation) and CFI (Comparative Fit Index) were considered for the evaluation of the goodness of fit of the models. Values below 0.08 for the RMSEA and values above 0.90 for the CFI were considered acceptable [59,60,61]. 

To determine the existence of differences between girls and boys in the tasks of the MOBAK 5-6 test and in the items of the SEMOK questionnaire, Student *t*-test for independent samples was used (*p* < 0.05). Cohen’s *d* score was quantified to analyze the effect of size of the comparisons, in this line, a *d*-value > 0.8 indicated a large effect, 0.8–0.5 a moderate effect, 0.5–0.2 a small effect, and <0.2 a trivial effect [62].

## 3. Results

### 3.1. Structural Model of the Relationship between Actual and Perceived Motor Competence

**Figure 1:**
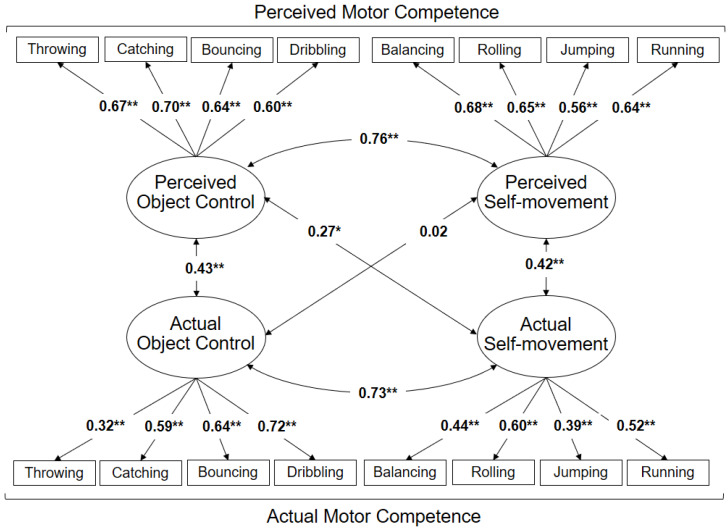
Relations between the factors of actual and perceived motor competence (MC) (model 1). * Significant differences at the level of *p* ≤ 0.05; ** Significant differences at the level of *p* ≤ 0.001.

Regarding the relationship between the factors of actual MC and the factors of perceived MC, the confirmatory factor analysis presents an acceptable adjustment index (*χ*^2^ = 192.81; *df* = 98; *p* < 0.001; *CFI* = 0.90; *RMSEA* = 0.05), confirming the 4-factor model and revealing a moderate correlation between actual and perceived MC (Figure 1). The correlation coefficient between actual and perceived object control factors is *r* = 0.43 (*p* < 0.001), while for the actual and perceived self-movement factors it is *r* = 0.42 (*p* < 0.001). While the correlation between perceived object control and actual self-movement is significant (*r* = 0.27, *p* = 0.008), the correlation between perceived self-movement and actual object control is not (*r* = 0.02, *p* = 0.781). The factor reliabilities (FR) are overall satisfactory in the majority of the factors (MOBAK object movement: FR = 0.66; MOBAK self-movement: FR = 0.58; SEMOK object movement: FR = 0.75; SEMOK self-movement: FR = 0.61).

### 3.2. Structural Model of Relationship between Actual and Perceived Motor Competence According to Gender

The multigroup model 2 combined two baseline models for boys and girls. The parameters were released for estimations. The factorial structure was kept equal for boys and girls. The model fit was satisfactory (*χ*^2^ = 332.8; *df* = 224; *p* < 0.01; *CFI* = 0.89; *RMSEA* = 0.046) and demonstrated that the factor structure is (configural) invariant between boys and girls.

Afterwards, we tested in multigroup model 3 whether the relation between the manifest and latent variables (factor loading) for boys and girls was the same (factorial invariance). The model fit of this model was also satisfactory (*χ*^2^ = 337.6; *df* = 236; *p* < 0.01; *CFI* = 0.90; *RMSEA* = 0.043) and slightly better than model 2. 

**Figure 2:**
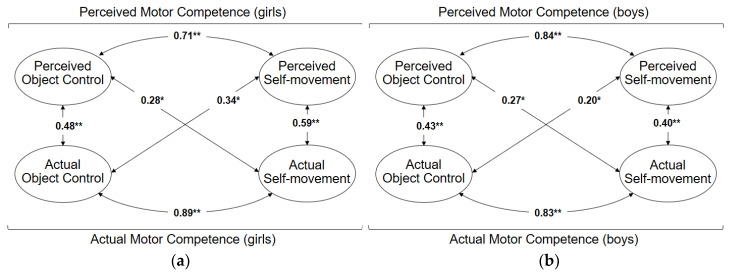
Relations between the factors of actual and perceived MC (model 3) (**a**) for girls; (**b**) for boys. * Significant differences at the level of *p* ≤ 0.05; ** Significant differences at the level of *p* ≤ 0.001.

The difference test between model 2 (variant factor loading) and model 3 (invariant factor loading) showed no significant difference (*χ*^2^ [12] = 14.89, *p* = 0.25). This provides the proof that the factor loadings between boys and girls were similar, and thus the measuring model can be assumed to be identical. The intercorrelation between the latent factors in the structure model showed slightly different values for girls (Figure 2a) and boys (Figure 2b), but these differences were overall not significant (*χ*^2^ [6] = 8.07, *p* = 0.23).

**Figure 3:**
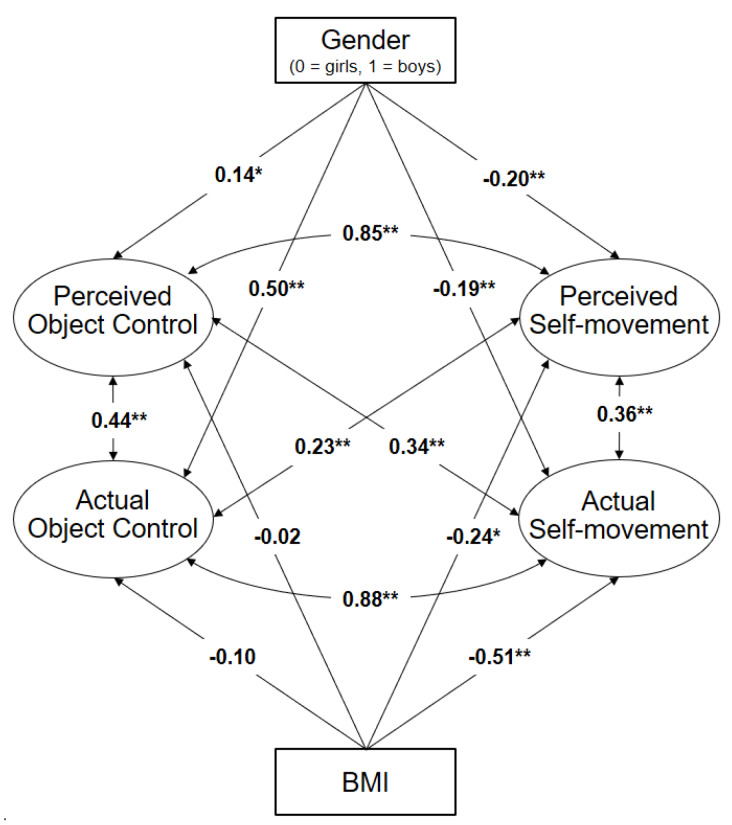
Confirmatory factor analysis with the covariate gender (model 4). * Significant differences at the level of *p* ≤ 0.05; ** Significant differences at the level of *p* ≤ 0.001.

Finally, we tested the gender differences in the latent factors between boys and girls and the association with BMI by calculating a confirmatory factor analysis with the covariate gender and BMI (model 4). The model still achieved acceptable fit values (*χ*^2^ = 194.8; *df* = 122; *p* < 0.01; *CFI* = 0.91; *RMSEA* = 0.038) and demonstrated that gender and BMI had significant influence on all four latent factors (Figure 3). Girls showed higher actual and perceived self-movement than boys, whereas boys performed and perceived higher in object control than girls. On the other hand, the BMI is only associated with the actual and perceived self-movement, not with the actual and perceived object control. The higher the pupils’ BMI was, the lower were their actual and perceived self-movement values.

### 3.3. Descriptive Statistics and Gender Differences in Actual and Perceived MC

Table 3 presents the descriptive values of the results regarding actual MC of each motor task of the MOBAK 5-6 test, differentiated according to the schoolchildren’s gender. Considering only the total values of the two factors, significant statistical differences were found in both, with the boys presenting higher values in object control than the girls and the girls presenting higher values than the boys in self-movement. These results on the manifest level of the sum values are in line with the results of model 4 on latent level. Regarding the detailed analysis of the items, the boys present higher values than the girls regarding the factor object control, with the exception of throwing, while the girls present higher values than the boys regarding the factor self-movement, mainly because of the item jumping.

Table 4 presents the results of perceived MC, for each item of the SEMOK questionnaire, according to the schoolchildren’s gender. The analysis of the total values of the two factors shows significant differences between boys and girls, with the boys indicating a higher perceived object control than the girls, and the girls indicating a higher perceived self-movement than the boys.

## 4. Discussion

The purpose of this study was to analyze the structure and correlations between actual and perceived MC in schoolchildren through structural models based on gender. The four-factor model showed an acceptable fit. Moreover, the question arose whether the underlying measuring model had a different factorial structure depending on gender. We tested this using nested grouping models. The factorial structure of the actual and perceived MC did not differentiate significantly between boys and girls. The measuring model was thus invariant in relation to gender. Therefore, the test instrument is suitable for showing the difference between boys and girls.

This study revealed interesting and novel evidence. On the one hand, boys showed a moderate association between actual and perceived MC regarding object control (*r* = 0.48) and self-movement (*r* = 0.40). On the other hand, girls showed a large correlation between actual and perceived MC regarding self-movement (*r* = 0.59), while the association in object control was moderate (*r* = 0.48). The capacity to recognize their MC in the late childhood has been confirmed in the analyzed global model of four factors (actual and perceived MC in object control and self-movement), which yields a moderate correlation between actual and perceived MC, both for object control and self-movement. Our results support the findings of Duncan and colleagues [18], Jaakkola and colleagues [63], and Lopes and colleagues [64] who also used specific measurements in the field of MC and where the perception of MC remains aligned with the actual MC.

So far, the studies that used aligned measurements of actual and perceived MC according to the specific MC domain have shown that the relationships were stronger in boys than in girls and in skills related to object control than to self-movement [22,65,66]. This study adds a new developmental view of the analysis of the association between actual and perceived MC: at the age of 10–13 the Chilean boys and girls are able to recognize their actual MC in object control at a moderate level. It should be noted that most studies have focused on the early childhood [17,18,20,21,23,27], an age at which they do not seem to possess the cognitive capacity to make accurate judgments and evaluate themselves [14,67] and at which they have limited possibilities to compare themselves with their peers [68]. During the transition from childhood to adolescence, the phase of life in which the participants of this study are, schoolchildren seem starting to be able to make accurate judgments of themselves [15]. During late childhood, they seem to have greater possibilities of social comparison than their younger peers, due to the time spent at school, facing different situations and putting their skills into practice, while recognizing the skill levels of others, which allows them to compare themselves and to know at what level they are [69].

Another interesting point to consider when the association between actual and perceived MC is under study is the relationship between the different types of FMS. Figure 1 presents for the whole sample only a relationship between perceived object control and actual self-movement (*r* = 0.27, *p* < 0.01). While analyzing Figure 2a (girls) and Figure 2b (boys), these cross-relationships are evidenced both between perceived object control and actual self-movement (girls *r* = 0.28, *p* < 0.01; boys *r* = 0.27, *p* < 0.01) and between perceived self-movement and actual object control (girls r = 0.34, *p* < 0.01; boys *r* = 0.20, *p* < 0.01). In essence, this could mean that by considering latent correlations without regard to gender, some results may be hidden. Therefore, it is important to consider correlates such as gender, since this way it is possible to obtain more accurate information about the relationship between actual and perceived MC.

Nevertheless, the literature on the relationship between actual and perceived gender-based MC has shown mixed results. On the one hand, some studies showed positive correlations mostly in object control and self-movement in boys and girls [18,19]; others found correlations in both object control and self-movement in boys, but in girls, these relationships only occur in self-movement [20]); others found correlations only in object control in boys but not in self-movement, neither in girls or in any case [21]. The results of this study are in accordance with those obtained by Herrmann and Seelig [19], where schoolchildren (boys and girls) in the last years of primary education were able to judge their actual MC in both object control and self-movement. The current study also showed that girls seem to be precise, being able to differentiate between the types of FMS, perceiving at which ones they are better (self-movement) and at which ones they have more difficulties (object control) in relation to their actual MC.

The comparative analyses seem to confirm what was found in previous studies according to gender and according to the type of MC. Regarding the actual MC, boys present higher values of MC in object control than girls, with a large effect size [5,18,41,70,71]. On the other hand, girls show a higher MC in self-movement than boys; although with a small effect size. These results support the findings of Liong and colleagues [21] and Strotmeyer and colleagues [41], but differ with the results of Barnett and colleagues [43,70] and Slykerman and colleagues [44] who found no gender differences in self-movement. With respect to perceived MC, the results support what was previously found by Barnett and colleagues [17,43], Estevan and colleagues [72], and Liong and colleagues [21], where boys self-reported higher values in object control than girls, although with a small effect size. In the same way, girls have a higher perception regarding self-movement than boys, with a medium effect size. These results in the field of self-movement partially align with those of Liong and colleagues [21], where girls indicated a higher perception than boys but whose difference was not significant. Other previous studies on younger children (5–11 years) found no differences in perceived MC in self-movement between boys and girls [14,43,44]. These findings at the end of childhood, where boys show more actual and perceived MC in object control than girls and where girls are perceived to be more competent in self-movement than boys, seem to confirm the trend in motor development according to gender associated with various sociocultural and environmental factors [23]. Boys, for example, seem to receive greater support and opportunities to participate in certain physical activities, especially in ball sports [19,41,48,73], while girls have lower levels of PA than boys [74], and their activities are related to individual sports such as dancing [48,73,75]. For this reason, it is necessary to develop intervention strategies that reduce these gaps and ensure equal participation of both genders. In this context, primary school teachers should take the self-perception and MC of boys and girls into account when they plan their classes in order to encourage their participation in motor proposals according to the type of MC [47].

Participation in PA might be deprived as a result of overweight and obesity during childhood [31]. Interestingly, in the current study BMI was only negatively associated with actual and perceived MC in self-movement but not in object control. Body self-movement and stabilization appears to be negatively associated with BMI because of the difficulty to control the body [29,30,31]. In addition, the effect size of the association between actual MC and BMI was large whereas in perceived MC and BMI it was moderate. During childhood, the construction of the association between BMI and MC is revealed to be larger in actual MC than in perceived MC. In line with Babic and colleagues [11] and D’Hondt and colleagues [29], special attention is needed for obese children, especially for those not practicing structured PA, in terms of fostering perceived and actual MC, respectively.

In the present study, domain-specific aligned and adjusted scales have been used to assess actual and perceived MC. The SEMOK questionnaire of perceived MC uses a pictographic scale, with graphic representations of each item. Despite the fact that the study focuses on the variable gender, both boys and girls appeared in the graphic descriptions, with the consequent influence of possible stereotypes depending on the type of item, such as in the conduction of the ball with the foot, where a boy appears. Another observation to take into account is the fact that the participants of this study are at the end of childhood, ad porta of adolescence, where the maturation status could affect their actual MC [76], an aspect that was not considered in our study and which would be interesting to analyze in future research. On the other hand, the level of PA practice of the participants was not analyzed in this study. As mentioned above, there is evidence to suggest that gender differences in MC are associated with the type of sport the children play, where boys are associated with team (ball) sports while girls are associated with individual sports [5,41]. This stereotyped social view may be an explanatory factor for differences in the perception of MC between boys and girls [77]. Therefore, in line with the motor development model proposed by Stodden and colleagues [2], it is suggested that future studies analyze the relationship between the type of PA or sport practice and the actual and perceived MC, using scales aligned and adjusted to the specific MC domain, differentiating the graphic representation according to gender. 

## 5. Conclusions

In conclusion, this study highlights the importance of the role that gender plays in the relationship between actual and perceived MC, with boys performing better and perceiving themselves as more competent than the girls in object control skills, while girls show better performances and perceive themselves as more competent in body control skills. At the same time, our results shore up the evidence on the negative association of BMI and actual and perceived self-movement, although in object control no association was found. The higher the students’ BMI was, the lower were their actual and perceived self-movement levels.

As reported in previous studies, there is still a lack of consensus regarding the relationship between actual and perceived MC, as well as regarding the role of gender. This study determines a positive association, both global and by gender, that supports the concept that at the end of childhood, the perception of MC tends to be more accurate [14]. Thus, the current study supports the existing evidence in the study of MC, indicating that the association between actual and perceived MC exists in both boys and girls, so that special attention should be paid to the development of FMS, as well as to the strengthening of the children’s sense of competence. In this line, the provision of support regarding perceived competence would offer a window of opportunity to generate adherence to the practice of PA, encouraging persistence and interest in activities in which the children perceive themselves as competent [2,15]. 

## Figures and Tables

**Table 1:** Description of the *Motoriche Basiskompetenzen* (MOBAK) 5-6 test items.

Item		Description
Object Control	Throwing	The child throws six juggling balls from a distance of 3.5 m at a target.
	Catching	The child throws a tennis ball at a wall from a scratch line at a distance of 3.0 m. The child catches the tennis ball directly from the air when it bounces back.
	Bouncing	The child bounces a basketball (size 6) back and forth through a marked corridor (8.0 × 1.1 m) with four obstacles of 0.7 m width, without losing the ball.
	Dribbling	The child dribbles a futsal ball (size 4) back and forth through a marked corridor (8.0 × 1.1 m) with four obstacles of 0.7 m width, without losing the ball.
Self-movement	Balancing	The child balances back and forth over an overturned long bench placed on a springboard, passing two obstacles taped to the bench (L: 17 cm, W: 10 cm, H: 12 cm) without touching them.
	Rolling	The child performs a forward roll, starting with a jump over a set-up banana box.
	Jumping	The child skips a rope for 20 s, changing rhythm after 10 s.
	Running	The child moves forward and sideways along a square (4.0 × 4.0 m) marked on the floor. While running forward, the child jumps through three evenly spaced hoops lying on the floor.

**Table 2:** Description of the Selbstwahrnehmung Motorischer Kompetenzen (SEMOK) questionnaire items.

Item		Description [Spanish Original]
Object Control	Throwing	I can hit a target with a ball. [yo puedo lanzar una pelota con precisión para darle a un objetivo en la pared]
	Catching	I can catch a ball securely. [yo puedo atrapar una pelota de tenis con seguridad]
	Bouncing	I can bounce a ball with my hands. [yo puedo conducir un balón de basquetbol]
	Dribbling	I can dribble a ball with my feet. [yo puedo conducir un balón de futbol]
Self-movement	Balancing	I can balance on a narrow beam. [yo puedo caminar hacia adelante y hacia atrás por una viga que se balancea]
	Rolling	I can perform a forward roll from a jump. [yo puedo hacer una voltereta hacia adelante con un salto previo]
	Jumping	I can jump the rope and change the rhythm. [yo puedo saltar la cuerda cambiando de ritmo]
	Running	I can change my rhythm when I’m running. [yo puedo correr cambiando de dirección]

**Table 3:** Descriptive results of actual MC according to gender ^1.^

Item/Factor	Girls		Boys		*t*	*df*	*p*	*d*
	*M*	*SD*	*M*	*SD*				
Throwing ^1^	0.51	0.65	0.57	0.65	−1.01	431	0.314	
Catching ^1^	0.31	0.62	0.86	0.84	−7.53	431	<0.001 **	0.75
Bouncing ^1^	1.05	0.84	1.41	0.75	−4.82	431	<0.001 **	0.45
Dribbling ^1^	0.44	0.72	1.00	0.81	−7.57	431	<0.001 **	0.73
Object Control ^2^	2.30	1.68	3.85	2.01	−8.52	431	<0.001 **	0.84
Balancing ^1^	0.76	0.82	0.61	0.79	1.81	431	0.070	
Rolling ^1^	0.50	0.80	0.63	0.86	−1.68	431	0.094	
Jumping ^1^	0.58	0.80	0.14	0.45	7.15	431	<0.001 **	0.68
Running ^1^	0.80	0.80	0.80	0.86	−0.10	431	0.920	
Self-movement ^2^	2.63	2.00	2.20	1.91	2.31	431	0.022 *	0.22

^1^ Range for each item: 0–2. ^2^ Range for each factor: 0–8. * Significant differences at the level of *p* ≤ 0.05; ** Significant differences at the level of *p* ≤ 0.001.

**Table 4:** Descriptive results of perceived MC according to gender.

Item/Factor	Girls		Boys		*t*	*df*	*p*	*d*
	*M*	*SD*	*M*	*SD*				
Throwing	3.93	0.98	3.80	1.21	1.17	458	0.244	
Catching	3.51	1.11	3.73	1.25	−1.94	458	0.053	
Bouncing	3.99	1.18	4.12	1.14	−1.19	458	0.233	
Dribbling	3.57	1.16	4.05	1.24	−4.26	458	<0.001 **	0.40
Object Control	3.75	0.79	3.93	0.88	−2.23	465	0.026 *	0.22
Balancing	3.64	1.23	3.23	1.38	3.35	458	0.001 **	0.31
Rolling	3.03	1.42	2.68	1.48	2.59	458	0.001 **	0.24
Jumping	3.86	1.32	2.81	1.46	7.98	458	<0.001 **	0.75
Running	4.47	0.87	4.37	1.05	1.16	458	0.248	
Self-movement	3.75	0.85	3.26	0.93	5.78	465	<0.001 **	0.55

Scale from 1 = totally disagree to 5 = totally agree. * Significant differences at the level of *p* ≤ 0.05; ** Significant differences at the level of *p* ≤ 0.001.
